# Novel coexisting mangrove-coral habitats: Extensive coral communities located deep within mangrove canopies of Panama, a global classification system and predicted distributions

**DOI:** 10.1371/journal.pone.0269181

**Published:** 2022-06-15

**Authors:** Heather A. Stewart, Jennifer L. Wright, Matthew Carrigan, Andrew H. Altieri, David I. Kline, Rafael J. Araújo

**Affiliations:** 1 Smithsonian Tropical Research Institute, Panama City, Republic of Panama; 2 Department of Biology, McGill University, Montreal, Quebec, Canada; 3 Department of Marine Biology and Ecology, Rosenstiel School of Marine & Atmospheric Science, University of Miami, Miami, Florida, United States of America; 4 Department of Natural Sciences, Sante Fe College, Gainesville, Florida, United States of America; 5 Department of Environmental Engineering Sciences, University of Florida, Gainesville, Florida, United States of America; University of Technology Sydney, AUSTRALIA

## Abstract

Marine ecosystems are structured by coexisting species occurring in adjacent or nested assemblages. Mangroves and corals are typically observed in adjacent assemblages (i.e., mangrove forests and coral reefs) but are increasingly reported in nested mangrove-coral assemblages with corals living within mangrove habitats. Here we define these nested assemblages as “coexisting mangrove-coral” (CMC) habitats and review the scientific literature to date to formalize a baseline understanding of these ecosystems and create a foundation for future studies. We identify 130 species of corals living within mangrove habitats across 12 locations spanning the Caribbean Sea, Red Sea, Indian Ocean, and South Pacific. We then provide the first description, to our knowledge, of a canopy CMC habitat type located in Bocas del Toro, Panama. This canopy CMC habitat is one of the most coral rich CMC habitats reported in the world, with 34 species of corals growing on and/or among submerged red mangrove aerial roots. Based on our literature review and field data, we identify biotic and abiotic characteristics common to CMC systems to create a classification framework of CMC habitat categories: (1) Lagoon, (2) Inlet, (3) Edge, and (4) Canopy. We then use the compiled data to create a GIS model to suggest where additional CMC habitats may occur globally. In a time where many ecosystems are at risk of disappearing, discovery and description of alternative habitats for species of critical concern are of utmost importance for their conservation and management.

## Introduction

Anthropogenic disturbances and climate change are impacting ecosystems globally and degrading their functioning by altering species composition and threatening species persistence [1–3]. This has led to a global biodiversity crisis with half a million species at risk of extinction in the coming decades [4, 5] and made conservation of remaining natural habitats critical to mitigate extinctions [5, 6]. Nearly half of the world’s population lives within 150 km of coasts, and as the human population continues to grow, unprecedented environmental pressures for land and resources pose great challenges to marine conservation [7–9]. Mangroves serve as biodiversity reservoirs, provide coastal protection, sequester 16% of the ocean’s carbon, and each year protect >15 million people and reduce property damage by >65 billion USD [10, 11]. However, tropical mangrove forests are considered one of the world’s most threatened ecosystems due to anthropogenic impacts, including conversion to aquaculture and agriculture, urbanization, exploitation of resources, and pollution [8, 9, 12]. By the end of the 20^th^ century, mangrove area had declined by 35% globally, and continued to be lost at a rate of 1–3% per year through the 2000s [13, 14]. Although deforestation of mangroves declined to 0.2–0.7% between 2000 and 2012, the synergistic interactions of natural and anthropogenic stressors has led to rapid and large-scale mangrove die-offs globally [15]. Tropical coral reefs are similarly important, harboring an estimated one third of all described marine species [16] and are also one of the most sensitive ecosystems to climate change [17]. Human activities have negative local impacts on coral reefs through sedimentation, nutrient runoff, pollution, and overfishing [18, 19], which reduce the resilience of corals to global stressors such as ocean warming and acidification [19]. A combination of these local and global factors has led to global mass bleaching events becoming increasingly frequent and severe, typically followed by reduced coral growth rates, decreased fecundity and recruitment, and high coral mortality [18, 20].

Corals and mangroves often co-occur in tropical coastal environments, and the scientific literature contains occasional observations of corals growing on or between mangrove roots in general biodiversity surveys [20–28] or as a dying coral reef is overgrown by mangrove forest with sea level fall [29–33]. Recently, however, there are an increasing number of empirical studies documenting nested mangrove-coral assemblages with corals establishing long-term and extensive communities within mangrove habitats [34–36] including locations in the Caribbean [37–45], Australia’s Great Barrier Reef [46], New Caledonia [47], Seychelles, and Sulawesi [48]. These nested mangrove-coral assemblages may be of evolutionary and ecological importance as potential genetic reservoirs, as climate refugia and as unique habitats created jointly by corals and mangroves.

Here we describe a novel coexisting mangrove-coral habitat in the Bocas del Toro Archipelago of Panama, which is one of the most extensive nested mangrove-coral assemblages ever reported, with many coral species growing on and among mangrove roots from the forest fringe extending up to 19 m into the mangrove canopy. In these habitats we found differences in coral community composition and morphology, as well as significant differences in abiotic conditions, among the mangrove canopy, mangrove edge, and shallow reef. These observations, along with prior published reports, led us to conclude that these habitats are ecologically distinct and thus in need of appropriate terminology to organize their study and identify general trends in their ecology. Moreover, because mangroves and corals are frequently referenced together in the literature (for example, searches of “mangrove AND coral” and “mangrove-coral” in Scopus yielded 18,660 and 210 hits respectively), and because, as we document in the present study, these systems show distinctive biotic and abiotic characteristics, we employ the term “coexisting mangrove-coral” (CMC) habitats to distinguish nested mangrove-coral assemblages from the adjacent coral reef and mangrove habitats that have traditionally been the focus of conventional work on these communities.

We reviewed the existing literature on co-occurrences of mangroves and corals, and added new data collected from our study system in Panama to synthesize a baseline understanding for further development of research on this topic. This study aims to (1) use field data and the existing literature to identify characteristics and conditions common to CMC habitats around the world; (2) describe a newly documented canopy CMC system in Bocas del Toro; (3) characterize different CMC habitat types to provide an easy-to-use classification system; and (4) predict where additional CMC habitats could occur worldwide using a GIS model.

## Materials and methods

### Review on CMC habitats

We conducted a review of mangrove-coral studies using Web of Science, Scopus, and Google Scholar using search parameters calibrated on 14 known articles (Bengtsson et al. 2019; Camp et al. 2016, 2017, 2018, 2019; Hernández-Fernández 2015; Macintyre et al. 2000; Rogers 2009, 2017, 2019; Rogers and Herlan 2012; Rützler et al. 2000; Yates et al. 2014; Wright 2019) on the mangrove-coral system (“original papers”), to discover how widespread CMC systems are reported in the literature. The most parsimonious search term which resulted in successfully detecting our 14 test articles were “mangrove* AND coral* AND scleractinian AND root”. Web of Science generated a list of 114 results, 4 of which were the original papers. Scopus detected 125 results, 5 of which were the original papers. Of all databases searched, only Google Scholar captured the full original target list and produced the most comprehensive results returning 1,920 candidate papers, which resulted in 966 after duplicates and citations were removed through the software Publish or Perish. In addition, we searched the references of all papers produced by the literature search. The last search date was January 3, 2021. One of us (HAS) examined search results to exclude publications that were outside the scope of our research or were simply referencing another paper that described mangrove-coral habitats leaving us with only 26 papers. We added ten publications from scientific reports, theses, and non-English literature based on our expert knowledge and communication with specialists for a total of 36 publications (S1 Table). Information extracted from the studies were: (1) location of observed mangrove-coral habitat, (2) type of mangrove-coral habitat (i.e., where the corals were found growing in relation to the mangroves), (3) mangrove species, and (4) coral species. From these data, we compiled a table with CMC habitats characterized by region, type, mangrove species, and number of coral species observed (Table 1), a table of the biotic and abiotic data collected in each study (S1 Table), and a table of the coral species observed growing in CMC habitats, separated by CMC type, region, and study (S2 Table). We intended to conduct a meta-analysis of existing literature to test whether our CMC habitat typologies fit within published research, which would allow us to identify the conditions necessary for corals to thrive within mangrove habitats and to predict where additional CMC systems may exist. However, we found a lack of environmental data in the CMC literature prior to 2014 (temperature n = 5, salinity n = 3, dissolved oxygen n = 2, turbidity n = 2, water flow n = 2, see S1 Table). Without a large enough dataset, a meta-analysis was not possible at this time.

**Table 1:** Coexisting mangrove-coral (CMC) habitat types and richness of mangrove and coral species by region.

Region	CMC Type	Mangrove Species Richness	Coral Species Richness
Caribbean Panama	Canopy^1^, Edge^1^^-^^3^	1	34
Caribbean Colombia	Edge^4^	1	1
Belize	Edge^5^^,^^6^, Inlet^6^^-^^9^, Lagoon^10^	1	19
Cuba	Edge^11^, Inlet^12^	1	14
Puerto Rico	Edge^13^^,^^14^	1	4
U.S. Virgin Islands	Edge^15^^-^^19^	1	35
Florida Keys	Edge^20^, Inlet^20^	1	6
Northern Red Sea	Lagoon^21^^-^^24^	3	1
Seychelles	Lagoon^25^	4	8
Sulawesi	Lagoon^25^	1	9
New Caledonia	Lagoon^26^, Inlet^27^	2	66
Great Barrier Reef	Edge^28^, Inlet^28^, Lagoon^28^^,^^29^	1	34

A list of mangrove and coral species by region, study, and CMC type is provided in S1 Table.

Coexisting mangrove-coral (CMC) habitats reported in existing literature and our study, indicating location of observations, type of CMC habitat, number of mangrove species corals were found growing within, number of coral species observed, and the source from which the information was obtained.

^1^ Present study and Stewart et al. 2021

^2^ Granek et al. 2009

^3^ Johnson 1992

^4^ Reyes and Campos 1992

^5^ Farnsworth and Ellison 1996

^6^ Rützler et al. 2004

^7^ Bengtsson et al. 2019

^8^ Scavo Lord et al. 2020

^9^ Scavo Lord et al. 2021

^10^ Macintyre et al. 2000

^11^ Hernández-Fernández 2015

^12^ de la Guardia et al. 2004

^13^ Almy et al. 1963

^14^ Jeffrey et al. 2010

^15^ Rogers 2009

^16^ Rogers 2017

^17^ Rogers and Herlan 2012

^18^ Buob 2019

^19^ Yates et al. 2014

^20^ Kellogg et al. 2020

^21^ Fishelson 1971

^22^ Por and Dor 1975

^23^ Loya 1976

^24^ Por et al. 1977

^25^ Camp et al. 2016

^26^ Camp et al. 2017

^27^ Maggioni et al. 2021

^28^ Stephenson et al. 1931

^29^ Camp et al. 2019.

### Description of CMC habitats in Panama

#### Bocas del Toro CMC habitat description

**Fig 1:**
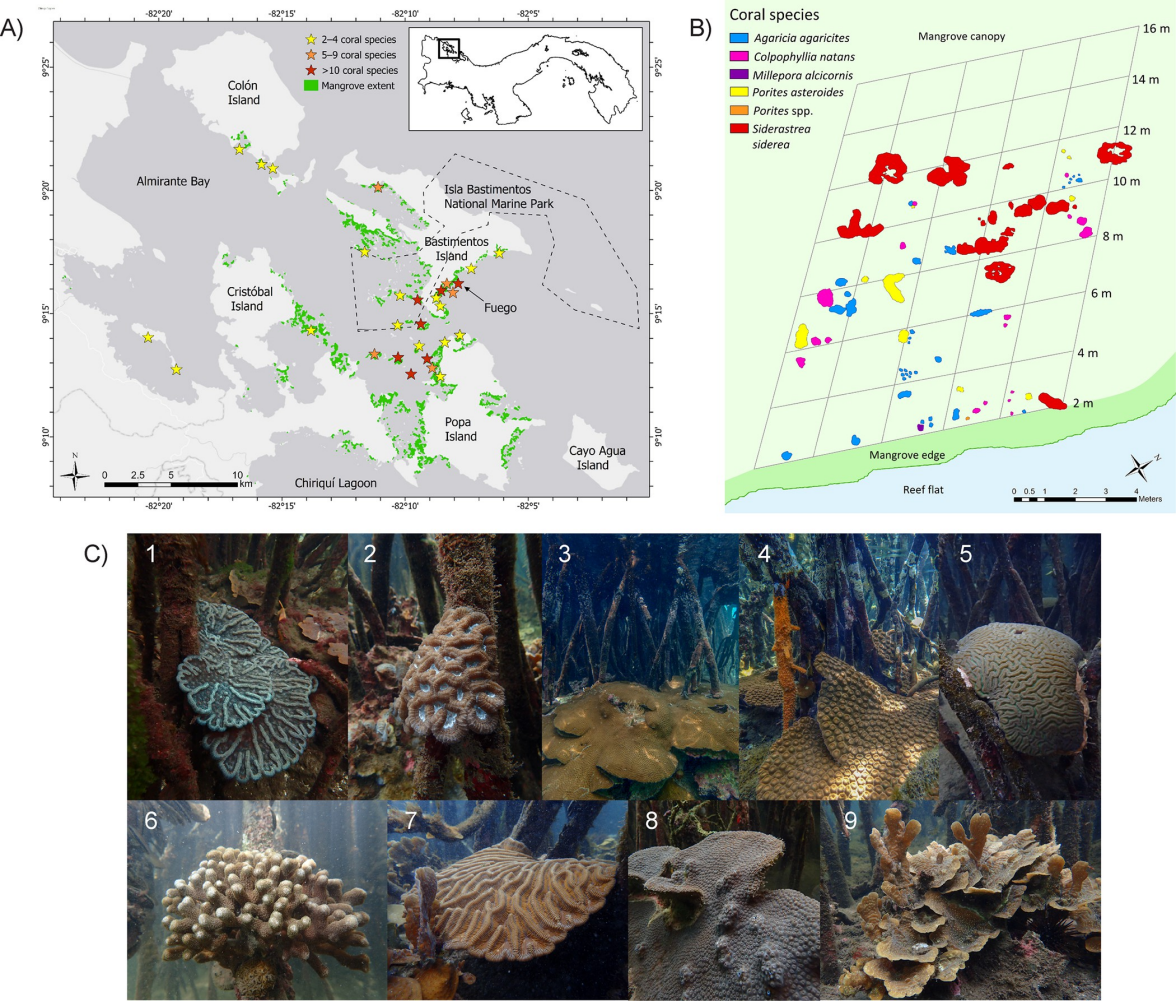
Coexisting mangrove-coral habitats of Bocas del Toro, Panama. (A) Coexisting mangrove-coral sites of Bocas del Toro with coral species richness, mangrove extent, and marine protected area (Isla Bastimentos National Marine Park). (B) mangrove canopy zonation map of coral colonies >5 × 5 cm at Fuego site. *Porites* spp. are branching species (*Porites porites*, *P*. *furcata*, or *P*. *divaricata*). (C) Mangrove coral growth forms and strategies (e.g., growing on or between roots). Coral species: (1) *Mycetophyllia aliciae* (2) *Isophyllia rigida*, (3) *Siderastrea siderea*, (4) *Montastraea cavernosa*, (5) *Diploria labyrinthiformis*, (6) *P*. *furcata*, (7) *Colpophyllia natans*, (8) *Orbicella faveolata*, (9) *Agaricia tenuifolia*.

The Bocas del Toro Archipelago of the Caribbean coast of Panama encompasses two large bays (Almirante Bay and the Chiriquí Lagoon), six large islands, a vast network of small mangrove cays, and mainland peninsulas fringed by mangroves (Fig 1A). The area has an estimated total mangrove cover of 28 km^2^, which is half of the total mangroves on the Caribbean coast of Panama [49, 50]. The vegetated fringe surrounding most islands of the archipelago is best characterized as a dwarf forest, dominated by red mangroves *Rhizophora mangle* rarely exceeding 2 m in height [51]. The islands of Bocas del Toro are surrounded by fringing coral reefs (<20 m deep) and shallow reef patches. Eighty-seven percent of the scleractinian coral species reported in Caribbean Panama are found in this area [49, 52]. Bocas del Toro has a characteristic zonation pattern of corals with *Porites furcata* dominating shallow areas, followed by *Agaricia agaricites* and *A*. *tenuifolia*, then massive corals like *Siderastrea siderea*, *Montastraea cavernosa*, *Orbicella franksi*, *Colpophyllia* spp., and *Stephanocoenia intercepta* on the reef slope [49].

#### Roving site assessments

Field observations in March 2017 suggested that distinguishing features of CMC habitats within Bocas del Toro from other mangrove habitats included: mangrove forest height ≤2 m, water inundation >1 m into canopy, water depth <2 m, open patches or channels within the mangrove canopy, high levels of water circulation, limited freshwater flow, clear water, and nearby reefs (within 5 m). Based on these observations and with the help of local boat captain Eric Brown, we conducted roving site assessments across the Bocas del Toro Archipelago in January 2018 and March 2019. These site assessments were used to determine how common CMC habitats were in this region and to evaluate how well these features correlated with coral richness of CMC habitats. Each roving site assessment covered approximately 100 m^2^, which took ~25–40 minutes depending on accessibility limitations due to root density in the mangrove habitat. During each rapid survey we recorded the number of coral species observed within the mangrove canopy, mangrove edge, and adjacent reef flat. At each site we recorded relative water clarity (e.g., visual distance), proximity to land development, freshwater inflow, and signs of pollution (e.g., garbage).

#### Canopy coexisting mangrove-coral map

In June 2018, we selected one of the CMC canopy sites with high coral richness (Fuego) on the eastern side of Bastimentos Island (N 09°16´23.1˝, W 082°07´53.1˝) to construct a 10 × 14 m grid by laying strings 2 m inland from the fringe/outermost aerial root, which was long enough to capture the distance to which corals extend into the mangroves (Fig 1B). This site was characterized by Holocene sediments [53], and a 50–500 m wide swath of mangrove forest around the edge of the island transitioning to tropical rainforest in upland areas. Moving offshore, the mangrove forest is adjacent to gradually sloping fringe reef (0.5–3 m depth). Due to the curvature of the island and mangrove canopy, we used 2 × 2 m parallelograms as cells within the grid to maintain the same distance (2 m) from the open water. Within each grid cell we recorded coral species richness, coral abundance (i.e., number and size of colonies), coral growth form (e.g., plating, mounding, encrusting), substrate on which coral colonies were growing, root density (quantified as the number of mangrove aerial roots including bifurcation), and water depth to establish zonation patterns. For comparison, we recorded coral richness, root density, and water depth on the adjacent mangrove edge and coral richness on the adjacent reef flat along a 50 m transect. To construct the mangrove canopy coral map, we measured and mapped the location of all colonies > 5 cm diameter within the grid. The GPS location of all four corners of the quadrat were taken in the field, uploaded to ArcGIS Pro v2.4.1 [54], and subsequently connected by drawing polylines in a newly created feature class to recreate the 10 × 14 m string grid. Next, a second feature class was created, and each coral was drawn as an additional polygon within that feature class.

#### Mangrove canopy coral colony monitoring and growth

Within the mapped grid, 17 of the largest coral colonies were tagged and numbered for further monitoring to determine growth and survivorship. We did this because skeletal measurements collected in conjunction with growth rate data can provide an estimate of coral colony age which is important in understanding the dynamics of the populations and demographics [55] within the mangrove habitat without destructive sediment coring. All colonies were measured using a fabric measuring tape and reference measurement points. We returned to the study site 15 months later (September 2019) to measure growth (i.e., change in surface area) and survivorship of the tagged coral colonies from 2018. We used simple elliptical and triangular geometric equations to calculate the area of the coral colonies [56].

#### Mangrove vs reef habitat comparisons

To get a better understanding of the mangrove-coral habitats compared to the adjacent reef habitats, we conducted a second survey in 2019 at the Fuego site expanding the original surveyed area. We recorded benthic percent cover within the mangrove canopy (>2 m inland from the fringe), the adjacent reef flat (>2 m seaward from the fringe), and the reef slope. This was done by placing 12 replicate 1 × 1 m quadrats along a 120 m transect in each coral habitat. Within each quadrat we also recorded the number of live coral colonies, noting coral species, colony size, and visible health ranking (i.e., visibly healthy, some bleaching/tissue loss, moderate bleaching/tissue loss, severe bleaching/tissue loss). Coral recruits were grouped by coral species as colonies <5 cm in size. To test for differences in benthic community composition between mangrove and reef habitats, we conducted a permutational multivariate analysis of variance (PERMANOVA) using the adonis2 function from the *vegan* package with the main effect of habitat type (mangrove canopy, reef flat, and reef slope). We then used a similarity percentage analysis (SIMPER) to determine which benthic groups had the greatest contribution to dissimilarity in the benthic community composition. For live coral analyses (coral species richness, diversity, and abundance of coral colonies), we used linear models to test for main effects and Tukey post-hoc tests were used for pairwise comparisons. Gaussian distribution was used in all regression models except for species richness for which we used the Poisson distribution.

#### Environmental conditions

At the Fuego site we recorded water temperature and light levels measured in lux every 15 min in the mangrove canopy (2–14 m from the fringe), mangrove fringe, and on the reef flat from mid-June to mid-July 2018 with data loggers (HOBO Pendant® Temperature/Light 64K Data Logger). We also measured light as photosynthetically active radiation (PAR) using a pair of spherical underwater quantum sensors (Li-cor LI-193) to take readings within the mangrove canopy and reef habitats simultaneously in July 2018 and September 2019. To analyze temperature and light data, we first checked for normality using qqnorm, then used two separate GLMs to test for main effects of position within CMC habitat (reef flat, mangrove fringe, and 2, 6, 8, 12, and 14 m into the mangrove canopy) and time of day as well as their interaction. If an interaction was found over 24 hours, a second GLM was ran at mid-day when temperature and light intensity were the greatest. If no interaction was found but significant differences were detected in one or more of the main effects, a Tukey post-hoc test was used for pairwise comparisons. We deployed YSI EXO2 multiparameter sondes in the mangrove canopy, mangrove fringe, reef flat, and reef slope in September 2019. These sondes recorded depth, water temperature, pH, turbidity, dissolved oxygen (ODO), salinity, specific conductivity, total dissolved solids (TDS), fluorescent dissolved organic matter (fDOM), and total chlorophyll (chlorophyll *a* and *b*) every minute from 10:30 am to 5 pm. To summarize and visualize the multivariate environmental data (water depth, pH, temperature, ODO, salinity, TDS, chlorophyll, and fDOM) collected in the mangrove canopy, mangrove fringe, reef flat, and reef slope, we used Principal Component Analysis (PCA) with the *ggfortify* and *factoextra* packages. All data were analyzed with R version 3.6.3 [57]. All data generated or analyzed during this study are included in the tables or supporting information. The scientific permit required to conduct this research was granted by the Republic of Panamá Ministry of the Environment (MiAmbiente).

### Coexisting mangrove-coral habitat types and definitions

We categorized CMC habitat types described in the literature and observed in our study into lagoon, inlet, edge, and canopy types based on physical setting and coral location in relation to the mangrove forest. We found these habitat types to fall amongst intersecting continuums of light and water flow. Non-CMC habitats, with full sun exposure and no shade provided by mangroves, at one end of the light continuum and mangrove interior canopy at the opposite end with full canopy cover at all times of day. Mangrove habitat type closely aligns with degree of water flow with open water, oceanic systems of fringe mangroves and overwash islands at one end and restricted water flow of interior systems and lagoons at the other end.

### Global mapping of CMC habitats

To identify areas where CMC habitats may occur globally, we explored the overlap between the global distributions of coral reefs (30 m spatial resolution) [58] and mangroves (25 m spatial resolution) [59, 60] using GIS. All files were uploaded to ArcGIS Pro v2.4.1 [54] as shapefiles using the World Behrmann Projected Coordinate System. Due to the large sizes of the datasets, all analyses were performed for 9 separate global regions, and then the merge and dissolve tools were used to combine these regions into one global extent. To find the greatest potential CMC habitat extent, we combined two global mangrove distributions [59, 60]. The mangrove extent from Spalding et al. (2010) and the coral reef shapefile were both created using data from 1999–2003. The mangrove data from Bunting et al. (2018) was available for 1996 and 2007, so we used the intersect tool in GIS to find the area that remained the same for these two time periods, resulting in mangrove extent for the time period of 1999–2003. Then, the erase, append, and dissolve tools were used to combine the Spalding et al. (2010) and Bunting et al. (2018) mangrove extents into one global shapefile. After creating the new mangrove extent, the overlap between the mangrove and coral shapefiles was analyzed using the intersect tool, resulting in a shapefile with potential CMC habitat extent. It is possible that some of these polygons overlapped with one another, resulting in duplicate CMC habitat area. To remove this potential issue, the dissolve tool was used to dissolve the boundaries between any adjacent or overlapping CMC habitat polygons. To compare how the model predictions of overlapping mangrove-coral habitats aligned with CMC observations, stars were added as reference points.

All presently reported CMC habitats in the literature occur at a shallow depth and are microtidal (≤2 m) [36, 44, 47, 48, 61, 62]. We therefore narrowed down the potential CMC habitat by areas with a tidal amplitude of ≤2 m, since a greater tidal amplitude would lead to prolonged air exposure of corals in this area. We hypothesized that areas with larger tidal amplitudes (e.g., Pacific coast of Panama, Mozambique Channel) have mangroves that are only periodically inundated with water, therefore limiting coral settlement and growth on/among the mangrove roots. We obtained global tide variables [63] and reclassified the raster file into two categories: ≤ 2 m and > 2 m. This reclassified raster was converted into a shapefile and the >2 m polygon was deleted from the shapefile, leaving only area with ≤2 m tidal amplitude. Finally, the clip tool was used on the overlap shapefile to obtain only the overlapping mangrove-coral extent that was within areas with ≤ 2 m tidal amplitude.

To validate the model, we crosschecked its output with known global CMC habitat locations. We then conducted ground-truthing within the Bocas del Toro Archipelago to test our model’s accuracy. We used the select by location tool to select all sites that were within 1 km of the overlap shapefile to compare model predicted CMC habitats to confirmed locations. For ground-truthing our observation of lower coral richness with proximity to freshwater input, we used the intermittent creeks shapefile from the Panama’s Hydrology Network layer of the Smithsonian Tropical Research Institute GIS Data Portal [64] in ArcGIS Pro to measure the distance of each CMC habitat site to the nearest creek endpoint. We then used survey data to compare coral richness of CMC habitats at varying distance from freshwater sources.

## Results

### Review on CMC habitats

There has been a rapid increase in publications involving CMC habitats, with 26 of the 36 relevant articles published since 2000 (Tables 1 and S1). The oldest reference to corals growing on mangrove roots we found was from the Great Barrier Reef Expedition 1928–1929 [65]. Stephenson et al. (1931) classified “mangrove swamp” as a region dominated by *Rhizophora* mangrove and other associated plants and animals. In the description of the mangrove sandy pools, the report mentioned “*Pocillopora bulbosa* flourishes here, sometimes growing on mangrove roots, and other living corals are species of *Acropora*, *Montipora*, *Millepora* and massive *Porites*, and Astraeids of more than one genus.” Additionally, in the description of the passages into the swamp, the report notes occurrences of several corals including *Porites*, *Cyphastrea*, and *Leptastrea*. Many of these older references are difficult to obtain electronically and do not appear in databases that do not contain historical records. However, a review of studies on “reef-top mangroves” of the Great Barrier Reef from the 1930s to the 1970s hypothesized that CMC ecosystems have been occurring since before the late 1920s despite the lack of earlier records [66]. The paradigm at the time was that this coexistence of mangroves and coral species was a brief timepoint in a “facultative successional sequence from corals to mangroves” as a results of the “complex interplay of reef growth, sea-level rise, and changing energy conditions over the last few thousand years” [66]. This may still be the case in many regions of the world [31, 32, 67], but since the coral colonies and mangroves are not coexisting, we would not consider these facultative habitats true CMC habitats.

Our search revealed that CMC habitats occur in virtually every tropical region of the world, with the highest documented number of coral species in locations in the Caribbean Sea, New Caledonia, and Australia’s Great Barrier Reef (Table 1). Although most articles broadly referred to “corals” without identifying species or abundance, we were able to extract coral species from 32 studies, including this study, and identified 130 species of corals and an additional 4 unique genera described as living in CMC habitats (S2 Table). The most described coral genera in decreasing order are *Porites* (n = 70), *Acropora* (n = 43), *Agaricia* (n = 27), *Millepora* (n = 22), *Siderastrea* (n = 22), *Orbicella* (n = 18), *Pseudodiploria* (n = 14), *Manicina* (n = 13), and *Favia* (n = 12). As for mangroves, New World locations are associated with *Rhizophora mangle*, while Old World localities had a greater variety of species within the genera *Rhizophora*, *Avicennia*, *Bruguiera*, *Lumnitzera*, and *Ceriops* (S2 Table).

The first study in our search including extensive abiotic data was published in 2014 [36], and in the time since, abiotic data have been published for only half of the locations where CMC habitats had been documented. An isotope study comparing the contributions of organic matter from mangrove detrital leaves, seagrass, macroalgae, and microalgae (phytoplankton and zooxanthellae) on corals on the mangrove edge, reef flat, and reef slope found that mangrove-derived nutrient incorporation by corals was present from 0.5 to 10 km from the mangrove forest and varied among coral species and sites [68]. Mangrove contributed organic matter was 20–40% for coral colonies growing directly on mangrove roots (i.e., *Agaricia tenuifolia* and *A*. *fragilis*) and 0–30% for corals on the reef flat and slope. A study of mangrove roots in Jardines de la Reina National Park, Cuba observed that 100% of CMC colonies were found in association with crustose coralline algae (CCA), primarily of the genus *Neogoniolithon*, suggesting that successful settlement of coral on submerged mangrove roots depends on the presence of CCA [45], although this association is not universal [41]. A qualitative assessment of the limited data from the literature suggests that CMC habitats require (1) a connection to the open ocean/open patches or channels within the mangrove stand, (2) submergence through all stages of the tidal cycle, (3) limited quantity or frequency of freshwater inputs, and (4) clear water [36–38, 41, 46, 48, 61]. Observed qualitative differences between mangrove sites with varying coral richness and diversity suggest a positive correlation with water flow and current speed [38, 41, 69]. However, the only studies that provided measurements of current speeds were the Stephenson et al. (1931) expedition which observed current speeds of 3 miles per hour or more in the mangrove passages in Australia and Rützler et al. (2004) which noted a current of 20 cm/sec in Hidden Creek, Belize. Also in Belize, Bengtsson et al. (2019) compared abundance of coral colonies among three contiguous bodies of water, a high-flow channel, a moderate-flow creek, and a low-flow mangrove pond and found coral species richness positively correlated with strength of water flow but flow was estimated based on stillness of water and effort needed by snorkelers to maintain their position. The lack of quantitative measurements limits further characterization of the importance of current speeds. We have compiled a summary of what type of biotic and abiotic data have been reported to date for CMC systems to serve as a reference for future studies (S1 Table).

### Description of CMC habitats in Panama

#### Roving site assessments

We observed corals growing within mangrove habitats at 29 sites across the Bocas del Toro archipelago, with coral species richness within the mangrove canopy varying by site: ≥10 species at 7 sites, 5–9 species at 5 sites, and 2–4 species at 17 sites (Fig 1A). Overall, we recorded 32 species of scleractinian corals within the mangroves, with 29 species occurring under the mangrove canopy and 20 species on the mangrove edge. Two milleporid coral species were also found under both mangrove canopies and edges. Comparatively, we observed 24 scleractinian species and 1 milleporid coral species on shallow, adjacent reefs, 13 scleractinian species on adjacent reef flats (<1 m depth) and 22 scleractinian species on adjacent reef slopes (>1.5 m depth) (Table 2). However, only a subset of those species were found in each respective habitat at a given site. Most observed corals were shallow (≤1 m), and 19 m was the furthest inland corals were found growing into the mangrove canopy. We found a positive association of coral richness with water clarity and size of mangrove area. Proximity to freshwater inflow, land development, and pollution appeared to be negatively associated with coral richness. We observed a decrease in coral species richness at multiple CMC sites between 2017 and 2019 at resampled sites, following nearby land development and the associated increase in sedimentation covering the mangrove roots (personal observation). Of the 29 CMC sites in Bocas del Toro, 4 were within 1.2 km of freshwater input (1 site off Colon Island, 1 site off Popa Island, 2 sites off Bastimentos Island), and all four sites were in the lowest species richness category. All sites with greater than 4 coral species were at least 1.8 km away from freshwater input.

**Table 2:** Coral species observed growing within coexisting mangrove-coral (CMC) habitats and adjacent reef in Bocas del Toro, Panama.

Coral Species	Edge CMC	Canopy CMC	Overall Mangrove	Reef Flat	Reef Slope	Overall Reef
*Agaricia agaricites*	X	X	X	X	X	X
*Agaricia fragilis*	X	X	X	X	X	X
*Agaricia humilis*		X	X		X	X
*Agaricia lamarcki*	X	X	X	X	X	X
*Agaricia tenuifolia*	X	X	X	X	X	X
*Colpophyllia natans*	X	X	X	X	X	X
*Diploria labyrinthiformis*		X	X		X	X
*Dichocoenia stokesi*		X	X		X	X
*Eusmilia fastigiata*	X	X	X	X		X
*Favia fragum*		X	X			
*Helioseris cucullata*		X	X			
*Isophyllia sinuosa*		X	X			
*Isophyllia rigida*		X	X			
*Madracis auretenra*					X	X
*Manicina areolata*	X	X	X	X	X	X
*Meandrina meandrites*					X	X
*Millepora alcicornis*	X	X	X	X	X	X
*Millepora complanata*	X	X	X			
*Montastraea cavernosa*	X	X	X	X	X	X
*Mussa angulosa*	X	X	X		X	X
*Mycetophyllia aliciae*		X	X			
*Orbicella annularis*	X		X		X	X
*Orbicella faveolata*	X	X	X		X	X
*Orbicella franksi*	X		X			
*Phyllangia americana*	X	X	X			
*Porites astreoides*	X	X	X	X	X	X
*Porites divaricata*		X	X	X		X
*Porites furcata*	X	X	X	X	X	X
*Porites porites*	X		X	X	X	X
*Pseudodiploria clivosa*	X	X	X		X	X
*Pseudodiploria strigosa*		X	X			
*Scolymia cubensis*	X	X	X		X	X
*Scolymia lacera*		X	X			
*Siderastrea radians*	X	X	X			
*Siderastrea siderea*	X	X	X	X	X	X
*Solenastrea bournoni*					X	X
*Stephanocoenia intersepta*		X	X			
Total species observed:	**22**	**31**	**34**	**14**	**23**	**25**

#### Canopy coexisting mangrove-coral map

Detailed mapping of 140 m^2^ of mangrove canopy shows 14 coral species growing in water depth ranging from 27 to 74 cm with high aerial root density (31–105 roots m^−2^) (Fig 1B). The nearby mangrove edge had 15 coral species in slightly deeper water (56–79 cm) and similar root density (15–129 roots m^−2^). In contrast, only six coral species were observed in the adjacent reef flat. At this site, corals were observed 15 m inland from the mangrove edge. The detailed map shows a zonation pattern of smaller corals (<10 cm) growing densely along the mangrove edge (0–2 m inland) with the occasional large colony (≥1 m diameter), followed by patches of corals with varying diversity and size further into the canopy (3–7 m inland), then transitioning into a zone with the greatest coral density, dominated by large (average surface area 1.3 m^2^), visibly healthy *Siderastrea siderea* colonies (8–12 m inland), and smaller, infrequent coral colonies beyond 12 m inland (Fig 1B). Within the mangrove habitat, corals grew on varied substrates (e.g., mangrove roots, bottom between roots, on other corals) and displayed variation in coloration and growth forms. On the mangrove edge both mounding and plating growth forms were observed, and coral colony colors resembled those of the adjacent reef. In the canopy encrusting and plating growth forms (Fig 1C) were predominant and there was more variation in colony coloration within species.

#### Mangrove canopy coral colony monitoring and growth

**Fig 2:**
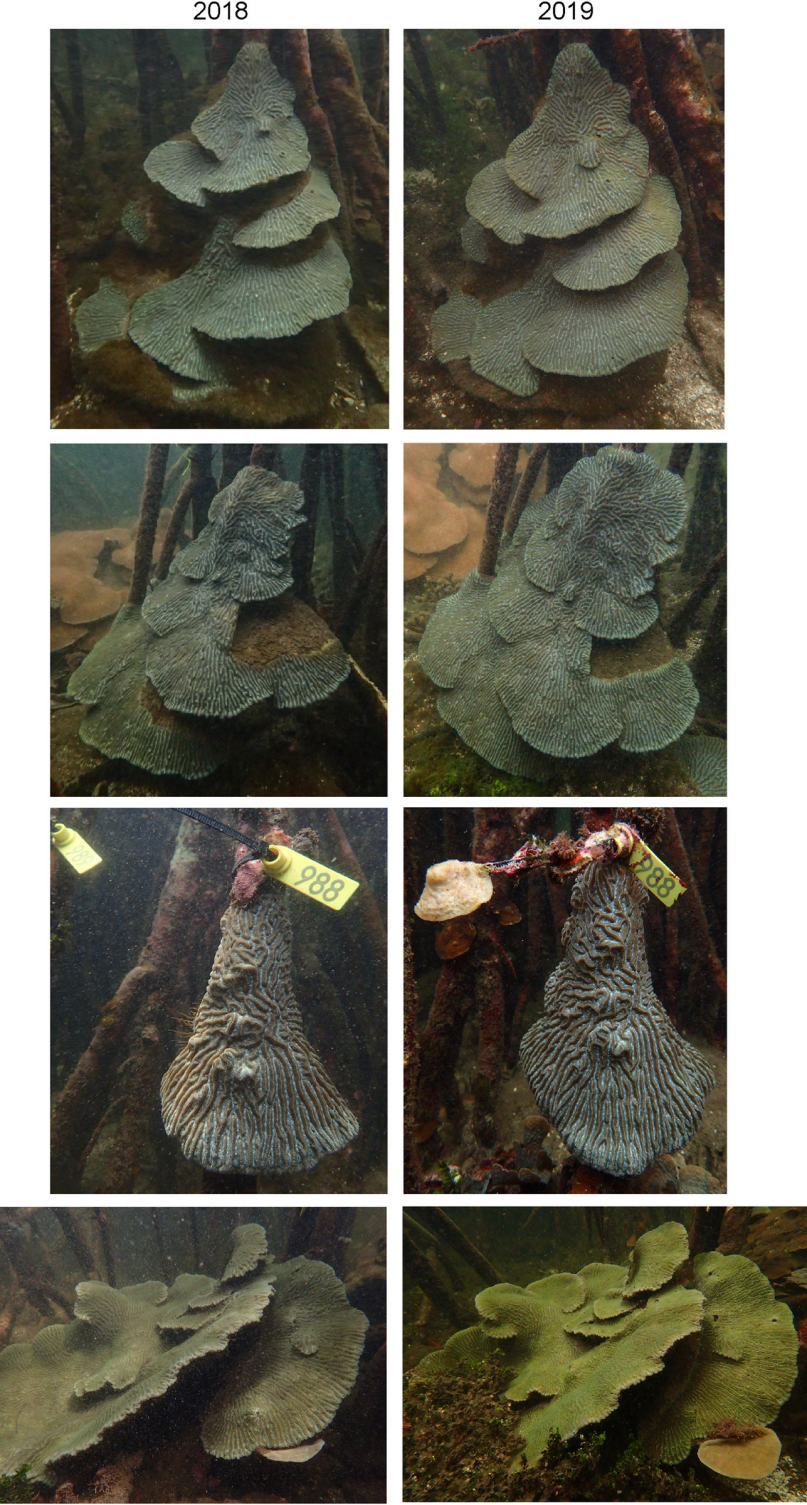
Coral colonies monitored for growth from June 2018 to September 2019. Photographs of four of the monitored coral colonies with year 1 on the left and year 2 on the right, showing how *Pseudodiploria* colonies had the capacity to regenerate plates after breakage.

Of the 17 individual coral colonies monitored for growth from June 2018 to September 2019, all showed sustained growth, except for a *Porites astreoides* colony, which decreased in area. The *Colpophyllia natans* colony went missing in 2019 (Table 3). Percentage of change in area varied with species and distance from the mangrove fringe. *Pseudodiploria clivosa* showed 5–17% increase in size at 6–10 m from the fringe and 132% increase 2 m from the fringe. It should be noted that *P*. *clivosa* grew in a plating morphology and these fragile plates would frequently break off from the colony or pull the entire colony off the mangrove root if they grew too wide. Successful *P*. *clivosa* wrapped around the mangrove roots and stabilized by spreading across multiple roots or towards the ground. Similarly, *Pseudodiploria strigosa* increased 14–41% at 4–8 m from the fringe and grew in a plating morphology but *P*. *strigosa* were found to both grow on the mangrove roots as well as on the coral rubble between the roots. Both *Pseudodiploria* species were able to regenerate plates after breakage (Fig 2). *Porites astreoides* colonies expanded over the monitored period but growth was difficult to calculate as the colony was dynamic with constant turnover of live corals bleaching or breaking and new recruits growing on the dead coral structure. The colony of *Agaricia fragilis* we monitored increased 80% in area and grew in a mixture of vertical and horizontal plates. Although individual plates showed signs of breaking over this period, overall, the colony grew. Recruits expanded to mangrove roots, but most of the colony grew on the coral rubble between the mangrove roots with the base largely covered in pink crustose coralline algae (CCA). The majority of the monitored coral colonies were *Siderastrea siderea* because they form the largest colonies within the mangrove with an average surface area of 1.3 m. The *S*. *siderea* colonies were most common 6 m or more from the mangrove fringe and increased 7–19%, although the largest colony at 16713 cm^2^ showed no change in area over the 14 months. All *S*. *siderea* colonies were healthy but some bleaching was observed in corals where mangrove roots rotted and broke off and landed on the coral as well as in areas where colonies of the zoanthid *Zoanthus pulchellus* overgrew colonies.

**Table 3:** Mangrove canopy coral colony growth.

Coral ID	Distance into Mangrove Canopy	Coral Species	Initial Area (cm^2^)	Change in Area (cm^2^)	Change in Area (%)
990	6–8 m	*Agaricia fragilis*	1925	1550	80
989	2–4 m	*Colpophyllia natans*	57	NA	NA
997	6–8 m	*Porites astreoides*	580	-10	-2
992	8–10 m	*Porites astreoides*	186	56	30
988	2–4 m	*Pseudodiploria clivosa*	90	119	132
985	6–8 m	*Pseudodiploria clivosa*	1785	112	6
998	6–10 m	*Pseudodiploria clivosa*	1655	89	5
986	8–10 m	*Pseudodiploria clivosa*	663	111	17
1000	4–6 m	*Pseudodiploria strigosa*	297	122	41
999	6–8 m	*Pseudodiploria strigosa*	971	135	14
991	6–8 m	*Siderastrea siderea*	15056	2046	14
996	8–10 m	*Siderastrea siderea*	8445	609	7
993	8–10 m	*Siderastrea siderea*	7106	1324	19
983	8–12 m	*Siderastrea siderea*	12417	1021	8
984	10–12 m	*Siderastrea siderea*	10295	227	2
994	10–14 m	*Siderastrea siderea*	12648	2241	18
995	12–14 m	*Siderastrea siderea*	16713	55	0

Tagged coral colonies monitored from June 2018 to September 2019. All corals showed sustained growth, except for one *Porites astreoides* colony (decrease in area). The *Colpophyllia natans* colony went missing in 2019.

#### Mangrove vs. reef habitat comparisons

**Fig 3:**
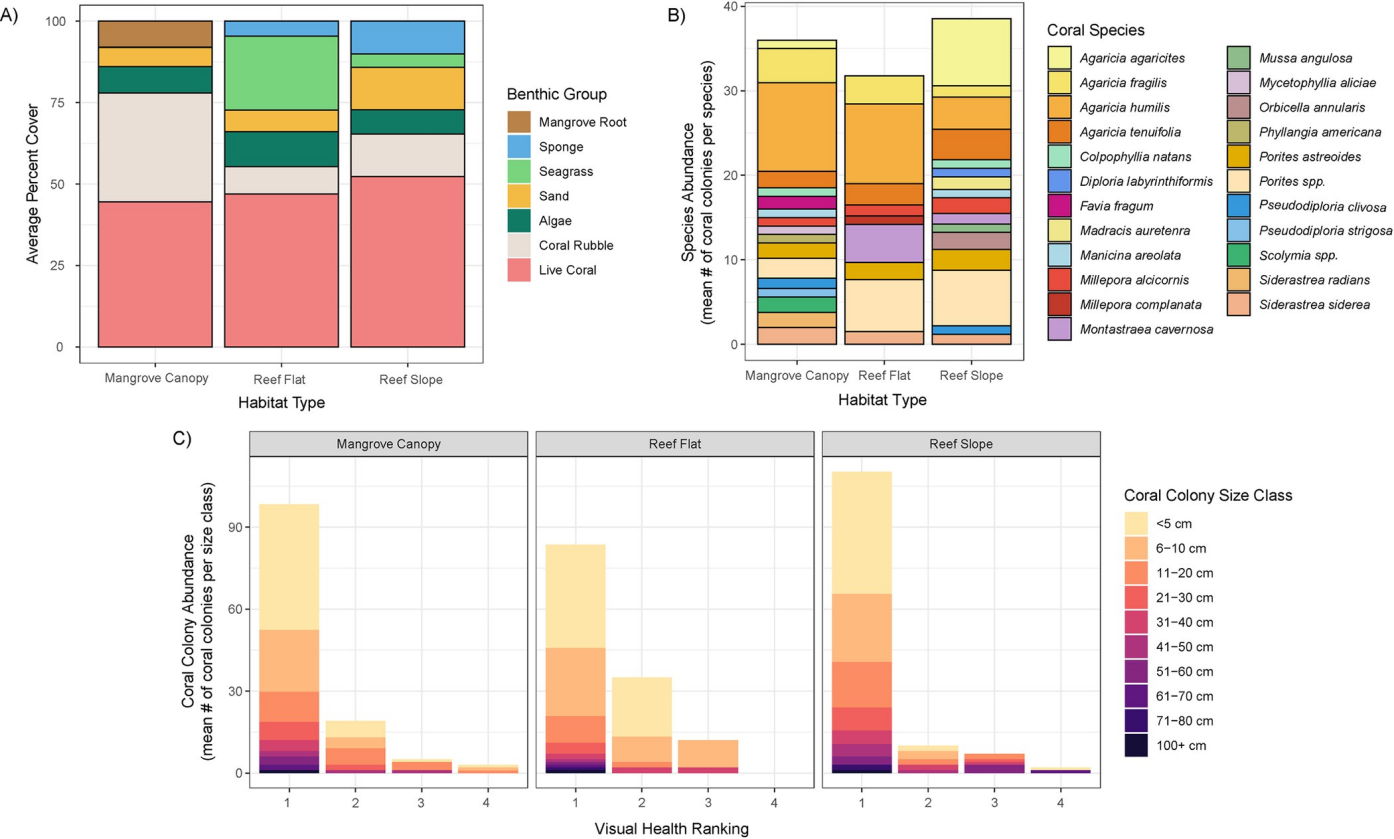
Coral community composition among coral habitats. (A) Average benthic percent cover of 12 replicate quadrats within three different coral habitats (mangrove, adjacent reef flat, and reef slope). There was no significant difference in the percent cover of live coral between habitats, but there was a significant difference in the percent cover of coral rubble with mangroves having 3x the amount of coral rubble of either reef habitat. (B) Average abundance of coral colonies by coral species among coral habitats. *Porites divaricata*, *P*. *furcata*, and *P*. *porites* are combined under *Porites* spp. (C) Average abundance of coral colonies by coral colony size class and visual health ranking (1 = visibly healthy, 2 = mild bleaching/tissue loss, 3 = moderate bleaching/tissue loss, 4 = severe bleaching/tissue loss) among coral habitats.

Habitat type (i.e., mangrove, reef flat, reef slope) had a significant effect on the composition of benthic cover (P = 0.005). The benthic cover groups contributing to the greatest dissimilarity between habitat types were coral rubble, seagrass, and mangrove root, with the mangrove habitat having the greatest amount of coral rubble (Fig 3A). Live coral made up the greatest mean percent cover of all benthic categories across habitat types (43.67 ± 0.05 mangrove, 42.42 ± 0.06 reef flat, 48.35 ± 0.07 reef slope, mean percent cover ± SE). Although there was no difference in percent cover of live coral between habitat types (P = 0.771), we found significant differences in species richness (P = 0.036) and Shannon diversity index (P < 0.001). Tukey post-hoc tests showed significantly greater coral diversity within the mangrove canopy and on the reef slope than on the reef flat, but no difference in coral diversity between mangrove canopy and reef slope (S3 Table). Quantity of live coral colonies varied significantly with coral species (P < 0.001) and colony size (P < 0.001), but not with habitat type (P = 0.872, Fig 3B and 3C). The mangrove habitat had similar amounts of coral recruitment as both reef habitats, reflected by the number of coral colonies <5 cm (P = 0.917). Coral recruits most commonly belonged to coral genera *Agaricia* or *Porites*.

#### Environmental conditions

**Fig 4:**
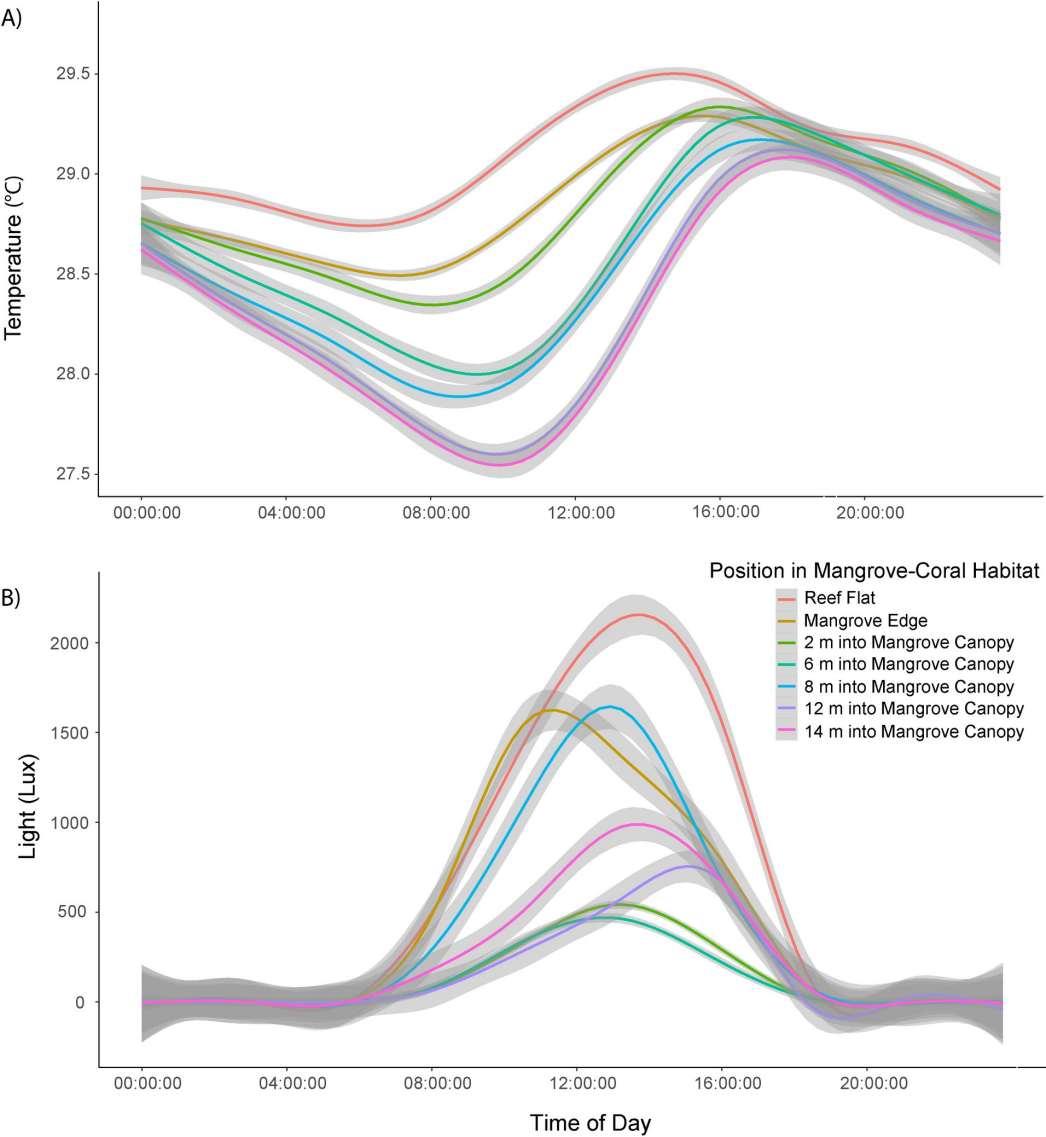
Daily (A) mean temperature and (B) mean light profiles. Data collected from June-July 2018 with 95% confidence interval (gray) of positions within mangrove-coral habitat on the reef flat (red), mangrove edge (gold), and mangrove canopy increasing every 2 m into the canopy from 2 m (green), 6 m (teal), 8 m (blue), 12 m (purple) to 14 m (magenta). Average water temperature was greatest on the reef flat and progressively decreased with depth into the mangrove canopy.

We found an interactive effect of distance from the reef and time of day on average water temperature (P < 0.001) with diel water temperature range increasing in variability with distance from the reef flat and peak separation in temperatures between 09:00 and 12:00 (midday) (Fig 4A). To better understand this separation, we ran a second general linear model (GLM) on average midday temperatures and found that the reef flat and mangrove edge were warmer than the mangrove canopy but there was no difference between the mangrove edge and reef flat, nor 2 m into the mangrove canopy (S4 Table). Maximum temperatures recorded were 31.2°C, 30.9°C, and 31°C on the reef flat, mangrove edge, and within the mangrove canopy, respectively. There was a similar interactive effect of distance from the reef flat on average light (P < 0.001) with the greatest diel range being on the reef flat and peak light intensity across areas occurring between 10:00 and 16:00. Unlike temperature, light intensity did not follow a clear pattern. The reef flat had both the greatest mean and maximum light levels, but light within the mangrove canopy was extremely patchy and did not correlate with distance from the reef (Fig 4B). Mean photosynthetically active radiation (PAR) measurements recorded at midday (11 am–1 pm) from 2018 and 2019 showed PAR levels to be 3.5–5.5 times greater on the reef flat than within the mangrove canopy. Mangrove PAR levels were 203 ± 80, 838 (mean ± SE and maximum) and 225 ± 28, 323 μmol m-2 s-1 in July 2018 and September 2019 respectively. Reef flat PAR levels were 729 ± 118, 1311 and 1205 ± 104, 1833 for the same time periods.

**Fig 5:**
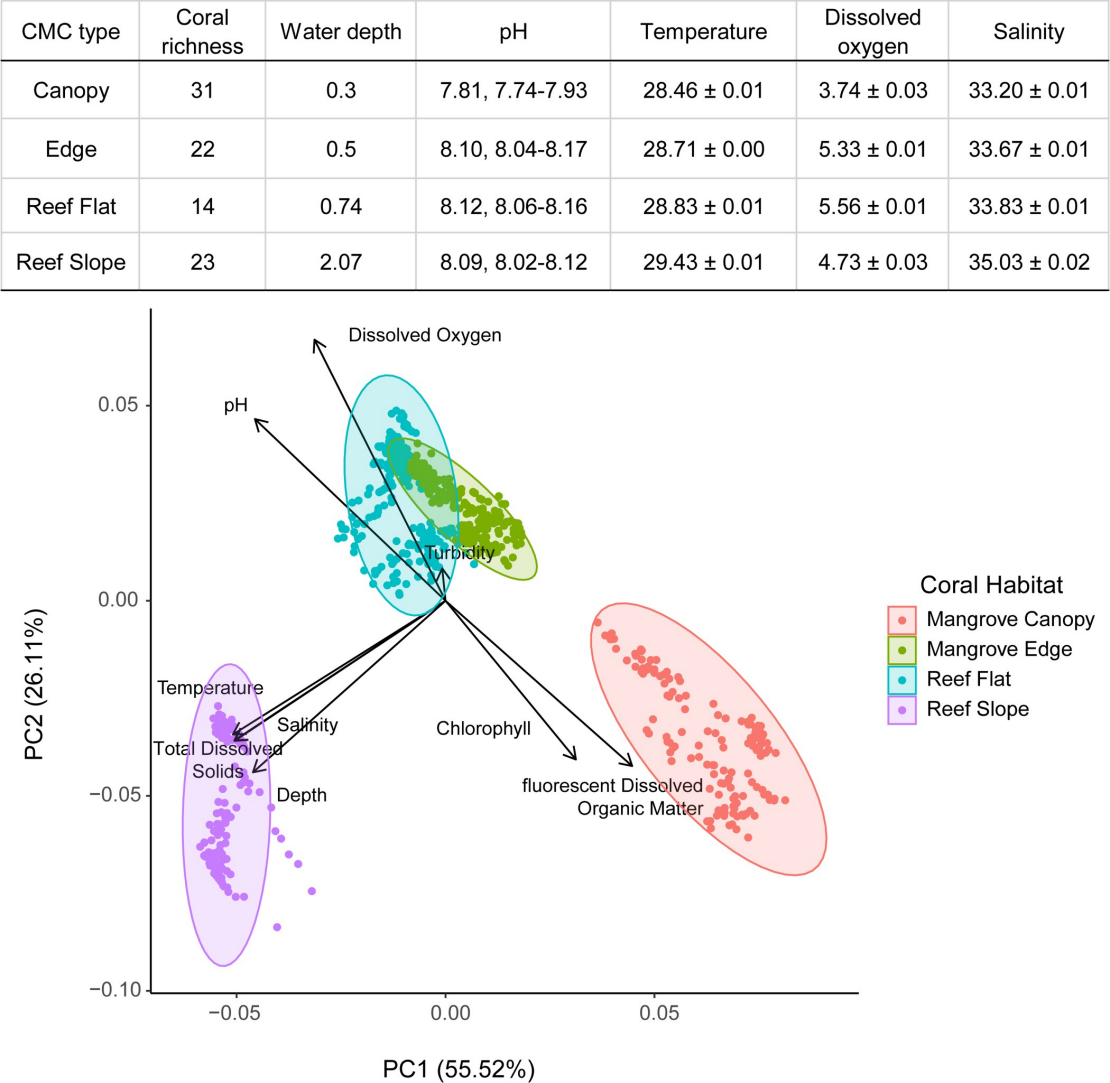
Abiotic distinction of coral habitats in Bocas del Toro, Panama. Principal Component Analysis of chlorophyll, fluorescent dissolved organic matter (fDOM), temperature, salinity, total dissolved solids (TDS), dissolved oxygen (ODO), and pH over four coral habitats in September 2019. Values given as mean ± SE except pH which is mean and range values. Units are meters for water depth, °C for temperature, mg/L for dissolved oxygen, and PSU for salinity.

Our PCA analysis found that within the CMC habitat, the mangrove edge and canopy separated as being distinct based on pH, ODO, turbidity, chlorophyll, and fDOM; meanwhile, the mangrove edge and reef flat were similar to each other (Fig 5). The mangrove canopy had the shallowest depth, and lowest levels of ODO, pH, temperature, and salinity (Fig 5), but the greatest average levels of chlorophyll and fDOM. The proportion of variance explained by PCA1 was 55.52% and 26.11% by PCA2, with a cumulative proportion of variance explained of 81.63% (Fig 5).

### Coexisting mangrove-coral habitat types and definitions

**Fig 6:**
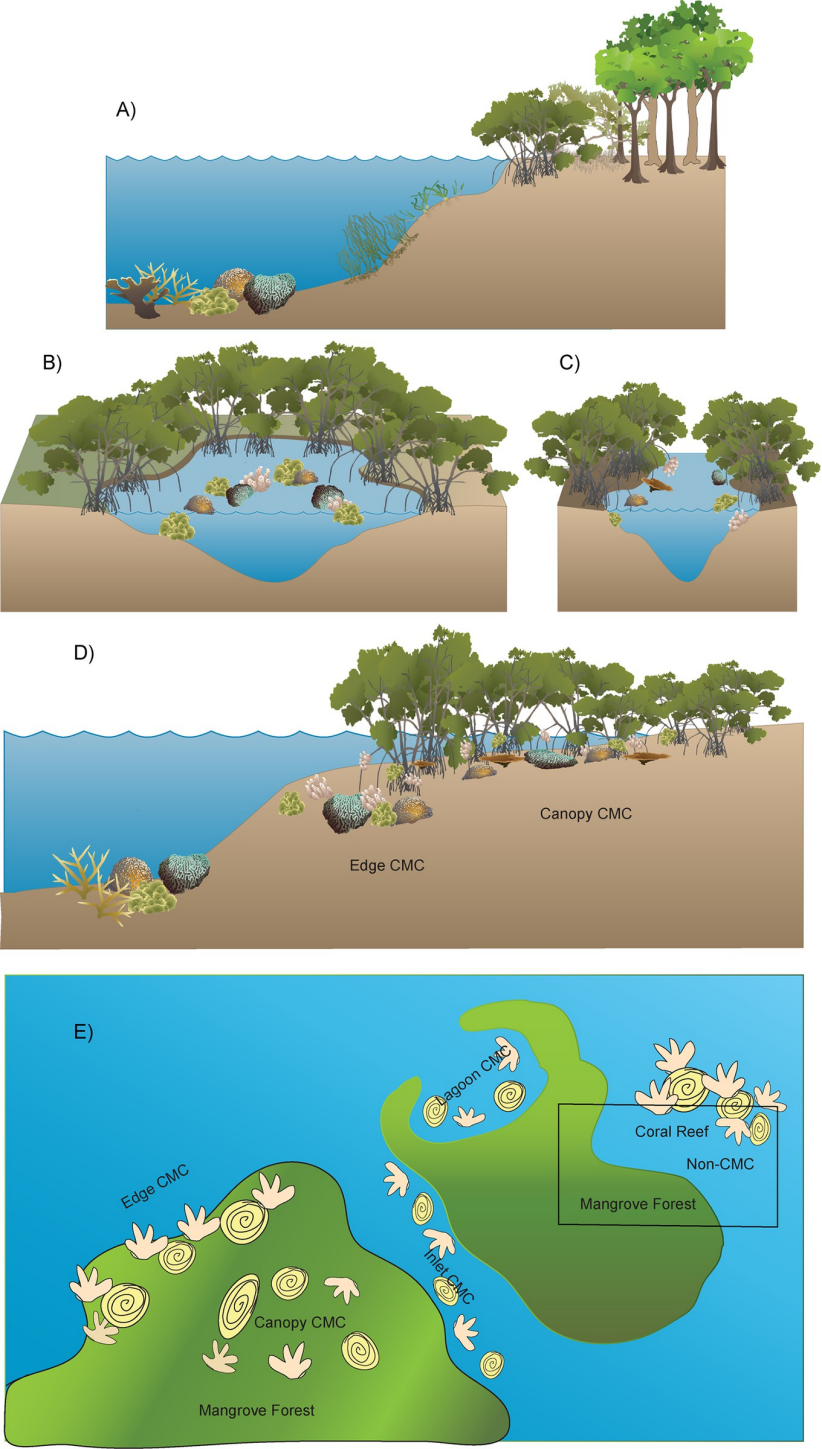
Coexisting mangrove-coral (CMC) assemblage types. (A) Non-CMC: discrete adjacent assemblage of coral reefs and mangrove forests. (B) Lagoon CMC: corals along semi-enclosed mangrove lagoons outside the shade of the mangrove canopy, but close enough for biogeochemical influence. (C) Inlet CMC: corals along mangrove channels partially under shade. (D) Edge CMC: partially or completely shaded corals grow on and around mangrove roots along the fringe of the mangrove and Canopy CMC: corals grow completely under the mangrove canopy with the lowest light among CMC habitats. (E) CMC habitats relative to each other. Graphics created by HAS with vector files from Tracey Saxby, Dieter Tracey, and Joanna Woerner Integration and Application Network, University of Maryland Center for Environmental Science (ian.umces.edu/imagelibrary/).

Based on field data and the current CMC literature, we identified the following CMC habitat types which we characterized based on physical setting and location of coral relative to the mangrove forest:

1. *Non-CMC habitat*: Adjacent assemblage of coral reefs and mangrove forests where these ecosystems create discrete zones and there is no influence of canopy shade on the reef (Fig 6A). A non-CMC habitat corresponds to the classic zonation pattern in which mangroves are near, but not overlapping with, a coral reef, oftentimes with seagrasses in between them. Common in the tropics, especially in areas with large tides, such as the Tropical Eastern Pacific, as well as in the Caribbean Sea.
2. *Lagoon CMC habitat*: Corals grow within lagoons that are semi-enclosed by mangroves and have restricted water flow. In these habitats, corals are positioned outside the shade of the mangrove canopy during high noon (Fig 6B). Observed in the Caribbean, South Pacific (New Caledonia), Indonesia (Sulawesi), Indian Ocean (Seychelles), the Great Barrier Reef, and the Northern Red Sea.
3. *Inlet CMC habitat*: Corals grow along mangrove channels or creeks (e.g., between mangrove islands or cays, natural tidal channels or man-made canals) partially to fully under the shade of the mangrove canopy (Fig 6C). Occurs in the Caribbean region and the Great Barrier Reef.
4. *Edge CMC habitat*: Corals grow on and around the mangrove roots along the fringe of the mangroves partially or completely under the shade of their canopy. Edge CMC habitats are typically fringe mangrove or overwash islands exposed to open ocean and have unrestricted water flow unlike Lagoon or Inlet CMC habitats. This category may include portions of fringe reefs where corals are growing under the shade of mangrove canopies (Fig 6D). Observed throughout the Caribbean region and the Great Barrier Reef.
5. *Canopy CMC habitat*: This habitat encompasses the most interior mangrove-coral setting and is connected to Edge CMC habitats through a transition zone. Corals grow on and around mangrove roots, completely under the shade of the mangrove canopy. Prop roots from the mangrove canopy edge restrict water flow to this habitat, cutting off direct connection with the open ocean (Fig 6E). Only reported in Caribbean Panama in this study and Stewart et al. 2021.

A visualization of CMC habitats is shown in Fig 6F. From the scientific literature review, a global tally of 63 coral species were identified within Lagoon, 82 within Inlet, 42 within Edge, and 31 within Canopy CMC habitats with edge and lagoon habitats being most extensively studied at this time [35, 36, 41, 46–48]. CMC habitats typically occur in fringe mangrove forests characterized by the genus *Rhizophora* and have been found in many intertropical regions around the world (*see* Table 1).

#### Additional features of coexisting mangrove-coral types

For ease of use in the field, we have based CMC classifications on the physical location of corals in relation to the mangroves. In this section we provide physical characteristics that might be used to further identify each type and can be compared to data available elsewhere. Other aspects of CMC physical setting and ecology that we have identified as distinct among types include (a) current speed, (b) temperature, (c) salinity, (d) light, (e) dissolved oxygen and (f) pH. Based on data collected in Panama, the interior nature of the Canopy CMC habitat contributes to the lowest light, temperature, and salinity (33.2 PSU) among the various CMC habitats and greater levels of chlorophyll and dissolved organic matter than Edge CMC habitats. Based on the limited data in the current literature, Edge and Lagoon CMC habitats had similar average salinity ranges (32.2–36.8 and 32.5–36.2 respectively), while Inlet CMC habitat salinity averaged 36.3 PSU. Dissolved oxygen was the lowest in the Canopy (average 3.74 mg/L), followed by Lagoon, and Inlet, with Edge having the greatest dissolve oxygen levels (averaging 5.33–7.22 mg/L). Our literature review suggests that Lagoon CMC habitats have the lowest pH range (7.31–8.08), followed by Canopy CMC (7.74–7.93), Inlet (7.78–8.40), and Edge CMC habitat having the highest pH range (7.92–8.70).

### Global mapping of CMC habitats

**Fig 7:**
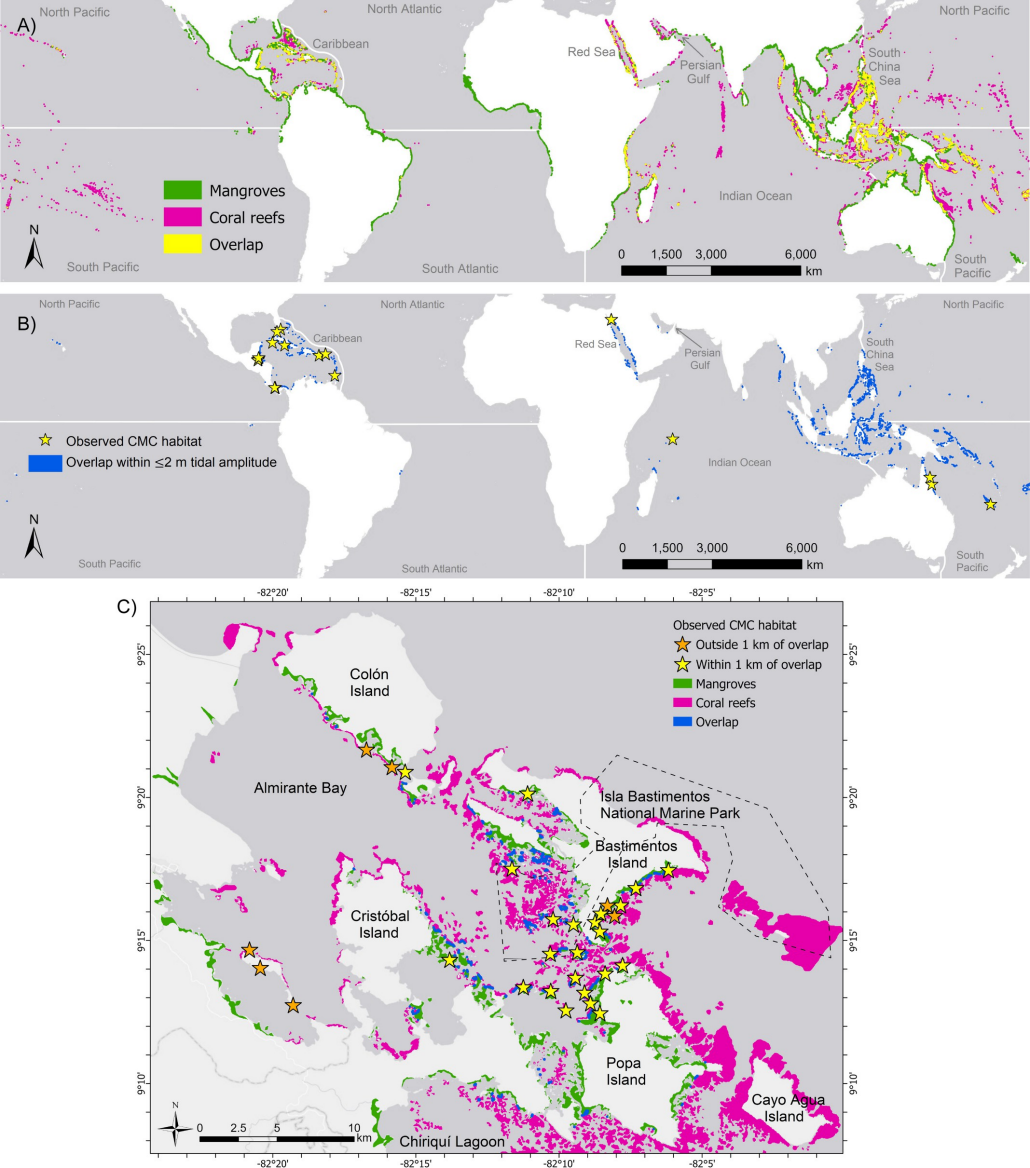
Potential global extent of coexisting mangrove-coral (CMC) habitats. (A) Global distribution of mangroves (green) and coral reefs (pink), showing overlap (up to 10 m annual average tidal amplitude) of their extents (yellow). (B) Microtidal overlap (≤2 m annual average tidal amplitude) wherein mangrove roots are fully submerged during both high and low tide. Stars represent CMC habitat locations obtained from literature review. (C) Ground-truthing of CMC model to validate accuracy based on crosschecking the output with known CMC habitat locations in the Bocas del Toro Archipelago. The model was able to predict 77% of our known CMC habitats within 1 km of the overlap shapefile.

The intersection of individual mangrove and coral reef extents resulted in an overlapping global area of 805 km^2^ (Fig 7A). Most of this overlapping area corresponded with microtidal regimes (i.e., ≤2 m annual average) which allow for mangrove aerial roots to be fully submerged through most tidal cycles so corals may grow with minimal air exposure, resulting in a potential global CMC habitat extent of 710 km^2^ (Fig 7B). The CMC habitat described in this study is in the Caribbean, where only 6.6% of the potential CMC habitat extent is found; the four locations with the greatest proportion of the worldwide potential CMC habitat are the South Pacific (44.8%), Red Sea (16.8%), North Pacific (13.8%), and South China Sea (10.8%, Table 4).

**Table 4:** Potential global extent of coexisting mangrove-coral habitats.

Region	Global Overlap (km^2^)	Global Overlap (%)
Red Sea	119.10	16.8%
Persian Gulf	0.44	0.1%
Indian Ocean	48.74	6.9%
South China Sea	76.76	10.8%
North Pacific	98.37	13.8%
South Pacific	317.89	44.8%
Caribbean	47.23	6.6%
North Atlantic	0.01	0.0%
South Atlantic	1.66	0.2%
**Total **	**710.20**	**100%**

Potential extent of mangrove and coral reef habitat overlap (within ≤2 m tidal amplitude; displayed in Fig 7), based on GIS analysis, divided into 9 global regions.

From our review and personal observations, we compiled a list of 30 CMC habitat locations throughout Bocas del Toro (Fig 7C). Of these 30 sites, 23 sites (77%) were within 1 km of the overlap model output. Three sites (10%) were not captured by the model around Isla Pastores because there was no mangrove coverage reported for that island, as well as no coral reef extent for the southwestern portion of the island. An additional 4 sites (13%)—two on Colon Island and two on Bastimentos Island—were not captured by the model (due to a lack of layer overlap) although the two habitat layers contained mangroves and nearby coral reefs in those locations.

## Discussion

Research on CMC habitats is rapidly developing due to its potential importance for marine conservation. Despite observations of corals growing on mangrove roots found sparingly in the scientific literature going back 90 years, there remains little known of the ecological parameters that define where these habitats exist. Natural history observations should be compiled with the existing quantitative data to help better describe these systems and understand their global importance. Potential environmental conditions that promote CMC habitats include a connection to the open ocean/open patches or channels within mangrove canopy, submergence through all stages of the tidal cycle, and proximity (~5 m) to coral reefs. On the contrary, conditions that prevent CMC habitat formation include land development, pollution, sedimentation, and freshwater in flow. We observed that land development, pollution, and sedimentation were negatively correlated with the number of coral species present in CMC habitats and frequently completely limited coral presence or survival. Freshwater in flow (e.g., proximity to a freshwater source such as rivers or creeks) seemed to consistently limit coral presence in our surveys, with the closest we found corals to freshwater input being 1.2 km. CMC habitats from the literature were described as without freshwater influence other than rain and subsoil water [38]. While some studies have mentioned the importance of current speeds [38, 41], we did not find consistency in current speed ranges that could be used as a criterion. In Panama we observed greater coral richness and abundance in windward mangrove areas relative to leeward sites, suggesting a potential importance of wave exposure. However, corals could be found in both habitats. The current literature is regionally skewed, with most published studies restricted to the Caribbean region [29, 35, 36, 38, 41, 43–45]. Until there is greater global representation, the current criteria we establish should be viewed as an evolving framework to inform potential metrics and to build upon. For example, in the Caribbean, corals in CMC habitats may need to be submerged through all stages of the tidal cycle, whereas in the Pacific where corals are more commonly exposed to air, tidal submergence may not be such a limiting factor.

The Bocas del Toro CMC habitats are primarily characterized by fringe forests and overwash islands in which *Rhizophora mangle* dominates [51], consistent with other locations throughout the Caribbean [36, 38, 41, 45]. We show that these nested assemblages are prevalent across the archipelago and support many coral species. The current study is the first, to our knowledge, to show that corals will expand far into the mangrove forest, often occurring up to 19 m inland from the mangrove fringe. Typical non-coral mangrove habitats have a thick benthic layer of detritus [60], however, in our benthic surveys of CMC habitats in Panama there was little to no detritus which may be an indicator of the amount of water flow and tidal flushing these unique systems have. The prevalence of plating and encrusting coral growth forms within the mangrove canopy as well as the color variation between coral colonies, suggest plasticity in growth form and photophysiology in response to the complexity of mangrove architecture where average light does not decrease with distance into the canopy and instead is patchy due to variation in canopy structure (e.g., sunflecks) and forest density. Since coral distribution within mangroves can be patchy [38, 44, 46], we suggest that coral distribution may be more closely related to light than temperature limitations. Darker pigment and flattened morphologies of corals are common in mesophotic reefs [70–72] as a physiological adaptation to low light conditions. These adaptations maximize light captured by increasing light-harvesting pigments and reducing the ratio of tissue to projected area for increased light capture efficiency [73–75], and could be utilized within the mangrove for the same purpose. Further, flattened morphologies may be a response of coral colonies to grow through lateral expansion rather than upward to avoid exposure at low tide [44]. However, the thin plate and cup forms of coral colonies may also limit growth within the mangrove habitat as these corals are extremely fragile and have been observed to break with root movement (e.g., swaying from boat wake). Based on our monitoring of mangrove corals, we found that corals that wrap around multiple mangrove roots or cement the root to the ground for stabilization were the more successful growth types, based on growth rate and survival.

Mangrove forests serve as critical habitat for many coral species [36, 38, 44], but can also be an extreme habitat, with low pH and highly variable temperature range, that selects for stress tolerant corals [20, 46–48, 76, 77]. Further research is needed to identify whether the roles served by mangroves are also reflected in CMC types, and if these are geographically distinct. Many of the most commonly observed coral genera found in CMC habitats are considered stress tolerant (*Siderastrea*, *Favia*) or weedy (*Porites*, *Manicina*, *Millepora*, *Agaricia*) coral taxa [78, 79], which have replaced more sensitive, competitive, branching coral genera on degrading reefs [79, 80]. These coral species have life-history characteristics that allow them to thrive in patch, chronically disturbed environments [81], which may be why they are so common in mangrove fringe communities. *Manicina aerolata* larvae, for example, attach to solid substrata (e.g., mangrove roots, coral rubble) which allows the free-living coral to colonize sediment-rich and dynamic habitats without being smothered. Eventually, the coral breaks free of the substratum to live unattached on the surface. Johnson (1992) proposed that large quantities of small coral colonies settle near the mangrove canopy and diffuse towards more turbid environments. Since most mortality occurs when coral colonies are small [81], that could explain why the percent cover of coral rubble was greater in the mangrove habitat than on the reef in our surveys in Panama. However, it must be noted that competitive (*Acropora*) and generalist (*Orbicella*) genera are among the six most frequently observed coral genera in CMC habitats. While coral reefs decrease in architectural complexity with the shifts from competitive species (e.g., acroporid corals) to stress tolerant and weedy taxa with simpler morphologies, CMC habitats may provide the structural complexity needed by larval fish and corals. We suggest that since corals are dependent on areal coverage of suitable substrate, the occupation of space by mangrove roots within CMC habitats may necessitate corals to utilize alternative morphologies to persist (Fig 1C), subsequently enhancing the mangrove habitat for fishes and invertebrates [40]. Stewart et al. (2021) found that CMC habitats supported greater coral species richness and diversity than the adjacent shallow reef, with no difference in the amount of live coral cover (30–36%) between the two habitats. Additionally, of six coral species experimentally transplanted into mangrove habitats from the reef, most species thrived in their new environment, demonstrating that these systems are an important habitat for corals particularly as the reef environment degrades.

While visually, coral habitats may be divided into reef and mangrove forest, abiotic factors on the edge of the mangrove canopy were more similar to the shallow reef than the mangrove canopy interior. The reduced water flow and greater shade provided by the mangrove canopy in the interior results in the lowest dissolved oxygen levels, temperature, salinity, and light and the greatest chlorophyll. The greater levels of fDOM in the mangrove canopy interior absorb more UV and visible radiation, altering the optical conditions of the environment which further impacts coral growth and photosynthesis. The zonation of coral species we observed in the mangrove canopy is most likely linked to the transition of abiotic conditions from the edge to the interior of the forest.

With an increasing rate of research emerging on nested mangrove-coral assemblages, consistent terminology is required to distinguish these systems from traditional adjacent coral reef and mangrove forest ecotones. We believe the terminology proposed here will help to identify the system being studied and aid in meaningful geographic comparisons, as has been done separately for mangrove forests and coral reefs [82, 83]. Just as fringe mangrove habitats may transition into basin mangrove forests, some of the CMC habitat types defined in this paper can transition into another.

Our GIS simulation based on tidal regime and mangrove/coral distribution suggested that the global extent of CMC communities could be substantial throughout the tropics. Although more knowledge is needed to refine global models, our model creates a baseline for the field to progress by suggesting which geographic locations should be the target of future studies. The Caribbean represented only 6.7% of the potential global extent of CMC habitats in our GIS analysis, despite leading in scientific reports. If further exploration is conducted across Pacific Ocean locations (where 58.6% of potentially suitable global CMC habitat occurs), we could perhaps better understand CMC ecosystem variability and the environmental conditions driving it. Improving our understanding of how coral and mangrove ecosystems interact is essential to determine the significance of CMC habitats for coral survival in the face of climate change and other anthropogenic impacts so that we can identify how to best conserve and protect these unique ecosystems.

### Future research

Despite knowledge of the existence of CMC habitats for at least 90 years, these systems are largely overlooked, and little is known about their function. Studies like Jeffrey et al. (2010) have focused on corals living within mangrove habitats compared to nearby reefs, yet only map and analyze the data from the reef, leaving us with a profusion of literature mentioning corals within mangroves as a footnote rather than studying the biodiversity of these habitats. When we refer to these papers, mention of corals “colonizing mangrove habitats” indicate CMC presence, but do not provide the necessary baseline to understand whether coral communities in CMC habitats are changing overtime or what role CMC habitats may play in the future. It is unclear if the logistics of collecting data in the mangrove habitat or accessibility are limiting factors or whether these systems have been ignored because they were viewed as unimportant or if these studies, focusing on other goals, did not have time or resources to explore mangrove-coral assemblages. That is why future research needs to move forward and make sure we are quantifying CMC coral community abundance, richness, health, and survival as well as measuring biogeochemical conditions within CMC habitats (e.g., diel and seasonal variations in water flow, seawater chemistry, salinity, PAR, and temperature) so that meta-analyses can be performed to improve our understanding of these systems and how CMC habitats may impact conservation of coral reefs. Additionally, further study into how different coral species use mangrove-derived organic matter could help understand coral community composition in these CMC habitat types. We also support Bengtsson et al.’s (2019) suggestion that future studies should specify whether coral species are attaching directly to mangrove prop roots or on some other substratum (e.g., coral rubble, peat) below the roots, as this distinction may be an important determinant in identifying suitable habitat for a given coral species. While we highlight the commonalities and distinctive characteristics of nested mangrove-coral assemblages, we are aware that we lack data on the health and functioning of CMC habitats in most parts of the world that may reveal important information about future trends of these mangrove-coral associations.

## Supporting information

S1 Table Results of review on coexisting mangrove-coral (CMC) literature. pCO_2_ = partial pressure of carbon dioxide HCO_3_ = bicarbonate, CO_3_^2-^ = carbonate ion, Ω_arg_ = aragonite mineral saturation state, and DIC = dissolved inorganic carbon. Winston 2007, MacDonald and Weis 2013, Wright 2019 all mentioned growing on mangrove roots but did not provide species information, so they were left out of this table.
(XLSX)

S2 Table Coral species observed growing within coexisting mangrove-coral (CMC) habitats. Summary of coral species observed in CMC habitats by region, CMC type, and reference paper. When only the coral genus was given, we used spp.
(CSV)

S3 Table Multiple comparisons of means of average mid-day temperature among distances from the reef. Estimate gives the difference between each treatment in the comparison. Significant p-values are in bold.
(XLSX)

S4 Table Tukey multiple comparisons of means for species richness and Shannon diversity among coral habitat types. Significant p-values are in bold.
(XLSX)

S1 Fig (JPG)
