# Supplementary figures and images for: Novel coexisting mangrove-coral habitats: Extensive coral communities located deep within mangrove canopies of Panama, a global classification system and predicted distributions
Source: PLoS One. 2022 Jun 15;17(6):e0269181. doi: 10.1371/journal.pone.0269181 (PMC9200167; doi:10.1371/journal.pone.0269181)

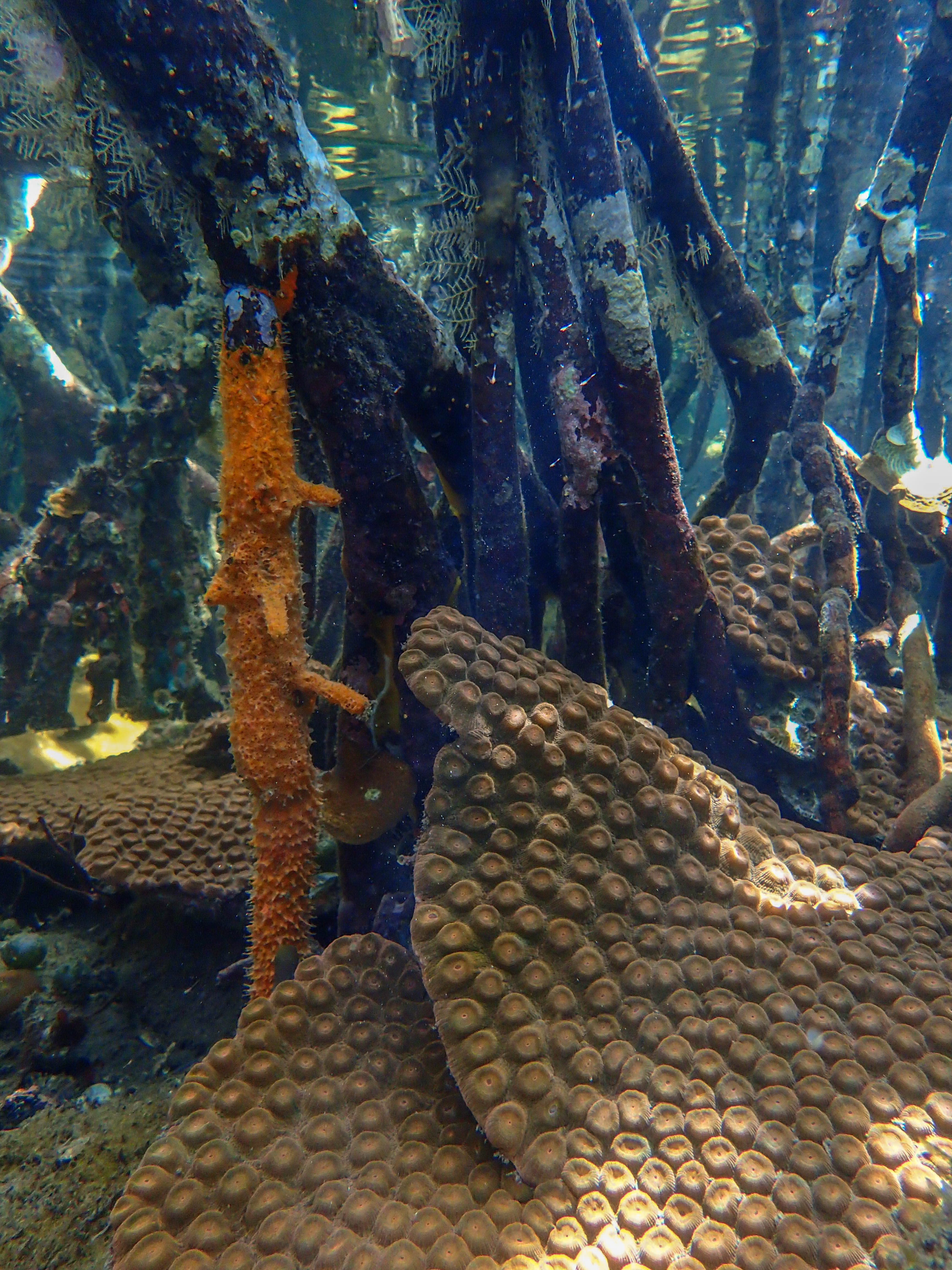

Supplement: S1 Fig — (JPG) [file pone.0269181.s005.jpg]
